# Mapping of lymphatic filariasis in loiasis areas: A new strategy shows no evidence for *Wuchereria bancrofti* endemicity in Cameroon

**DOI:** 10.1371/journal.pntd.0007192

**Published:** 2019-03-08

**Authors:** Samuel Wanji, Mathias Eyong Esum, Abdel Jelil Njouendou, Amuam Andrew Mbeng, Patrick W. Chounna Ndongmo, Raphael Awah Abong, Jerome Fru, Fanny F. Fombad, Gordon Takop Nchanji, Glory Ngongeh, Narcisse V. Ngandjui, Peter Ivo Enyong, Helen Storey, Kurt C. Curtis, Kerstin Fischer, Joseph R. Fauver, Daphne Lew, Charles W. Goss, Peter U. Fischer

**Affiliations:** 1 Parasites and Vector Biology research unit (PAVBRU), Department of Microbiology and Parasitology, University of Buea, Buea, Cameroon; 2 Research Foundation for Tropical Diseases and the Environment (REFOTDE), Buea, Cameroon; 3 Diagnostics Program, PATH, Seattle, Washington, United States of America; 4 Infectious Diseases Division, Department of Internal Medicine, Washington University School of Medicine, St. Louis, Missouri, United States of America; 5 Division of Biostatistics, Washington University School of Medicine, St. Louis, MO, United States of America; Ministère de la Santé Publique et de la Lutte contre les Endémies, NIGER

## Abstract

**Background:**

Mapping of lymphatic filariasis (LF) caused by *Wuchereria bancrofti* largely relies on the detection of circulating antigen using ICT cards. Several studies have recently shown that this test can be cross-reactive with sera of subjects heavily infected with *Loa loa* and thus mapping results in loiasis endemic areas may be inaccurate.

**Methodology/Principal findings:**

In order to develop an LF mapping strategy for areas with high loiasis prevalence, we collected day blood samples from 5,001 subjects residing in 50 villages that make up 6 health districts throughout Cameroon. Antigen testing using Filarial Test Strip (FTS, a novel platform that uses the same reagents as ICT) revealed an overall positivity rate of 1.1% and *L*. *loa* microfilaria (Mf) rates of up to 46%. Among the subjects with 0 to 8,000 Mf/ml in day blood, only 0.4% were FTS positive, while 22.2% of subjects with >8,000 Mf/ml were FTS positive. A Mf density of >8,200 Mf/ml was determined as the cut point at which positive FTS results should be excluded from the analysis. No FTS positive samples were also positive for *W*. *bancrofti* antibodies as measured by two different point of care tests that use the Wb123 antigen not found in *L*. *loa*. Night blood examination of the FTS positive subjects showed a high prevalence of *L*. *loa* Mf with densities up to 12,710 Mf/ml. No *W*. *bancrofti* Mf were identified, as confirmed by qPCR. Our results show that high loads of *L*. *loa* Mf in day blood are a reliable indicator of FTS positivity, and Wb123 rapid test proved to be relatively specific.

**Conclusions/Significance:**

Our study provides a simple day blood-based algorithm for LF mapping in loiasis areas. The results indicate that many districts that were formerly classified as endemic for LF in Cameroon are non-endemic and do not require mass drug administration for elimination of LF.

## Author summary

High prevalence of loiasis can confound the mapping efforts for lymphatic filariasis (LF) that use tests for circulating filarial antigen. In order to develop a LF mapping strategy for loiasis endemic areas, we screened day blood samples from 5,001 subjects residing in 50 villages in 6 health districts throughout Cameroon. Circulating filarial antigen (FTS) rates varied from 0.3 to 2.2% between health districts while *L*. *loa* microfilaria (Mf) rates varied between 0 and 46%. Around 0.4% of the subjects with no or less than 8,000 Mf/ml were FTS positive, while 22.2% of subjects with >8,000 Mf/ml were FTS positive. A *L*. *loa* Mf density of >8,200 Mf/ml was determined as the cut point at which positive FTS results should be excluded from the analysis. No FTS positive sample was positive for *W*. *bancrofti* antibodies as measured by two different point of care tests that use an antigen not found in *L*. *loa*. Night blood examination of the FTS positive subjects also showed a high prevalence of *L*. *loa* Mf with densities up to 12,710 Mf/ml, but no *W*. *bancrofti* Mf as confirmed by qPCR. Our results show that high *L*. *loa* Mf in day blood (> 8,200 Mf/ml) are a predictor for FTS positivity and rapid antibody tests specific for *W*. *bancrofti* can be used to confirm the absence of *W*. *bancrofti*. Our study provides a simple day blood-based algorithm for LF mapping in loiasis areas. The results indicate that many districts that were formerly classified as endemic for LF in Cameroon are non-endemic and do not require mass drug administration for elimination of LF.

## Introduction

Lymphatic filariasis (LF) is a neglected tropical disease that is targeted for elimination. A key intervention strategy is mass drug administration (MDA) in all endemic areas with a microfilaria (Mf) or antigen prevalence of at least 1%. The Global Program to Eliminate Lymphatic Filariasis (GPELF) has made substantial progress in a number of countries, but it lags behind in others, especially in countries in western and central Africa [1, 2]. In order to tailor a cost-effective and successful MDA program to country specific needs, accurate mapping of lymphatic filariasis distribution is a prerequisite. For most areas in sub-Saharan Africa, a combination of ivermectin with albendazole is used for MDA. However, in western and central Africa where the filarial parasite *Loa loa* is highly co-endemic, twice yearly MDA with albendazole is recommended [3]. The latter strategy has different dynamics for reduction of infection than ivermectin combined with albendazole, but was recently shown to be effective for the local elimination of LF [4].

There are little historical data on the distribution of LF in Cameroon. LF was assumed to be widespread, especially in the northern part of the country, but confirmed parasitological data were scarce [5]. A few studies report low prevalence of microfilaremia, not exceeding 1% [6, 7]. The presence of clinical disease, especially lymphedema, is considered to be a poor indicator for LF in Cameroon, because podoconiosis associated lymphedema is common in some areas [8–10]. In Africa, LF is exclusively caused be the filarial parasite *Wuchereria bancrofti* and detection of circulating *W*. *bancrofti* antigen in human blood using rapid diagnostic tests has largely replaced microscopic detection and identification of microfilariae (Mf) in night blood for mapping. While this powerful approach is facilitating the mapping of LF in many areas, several recent studies have shown that this strategy is not reliable in areas highly endemic for *L*. *loa* [11–13]. Individuals with high levels of *L*. *loa* Mf may contain sufficient cross-reactive antigens in their blood that can be detected by the antibodies used in the diagnostic tests for *W*. *bancrofti*. Another confounder is that *L*. *loa* Mf are sometimes found in moderate to high densities in night blood and could be easily misidentified by microscopy with nocturnally periodic Mf of *W*. *bancrofti* [11]. Given these findings, LF mapping results from *L*. *loa* endemic countries using mainly circulating filarial antigen tests must be interpreted with great caution [14].

The present study was designed to develop a simple and reliable algorithm for LF mapping in areas co-endemic for *L*. *loa*. We collected day blood samples from about 5,000 subjects from 6 health districts located in different ecological zones and performed blood smears alongside rapid filariasis antigen and antibody tests. We followed up the antigen positive subjects with night blood collection and performed blood smears as well as qPCR to detect *W*. *bancrofti* and *L*. *loa* DNA. The study answered the question whether positive antigen tests for *W*. *bancrofti* were due to cross-reactivity with *L*. *loa* and found no evidence for LF endemicity in any of the 50 study villages.

## Methods

### Study sites

**Fig 1:**
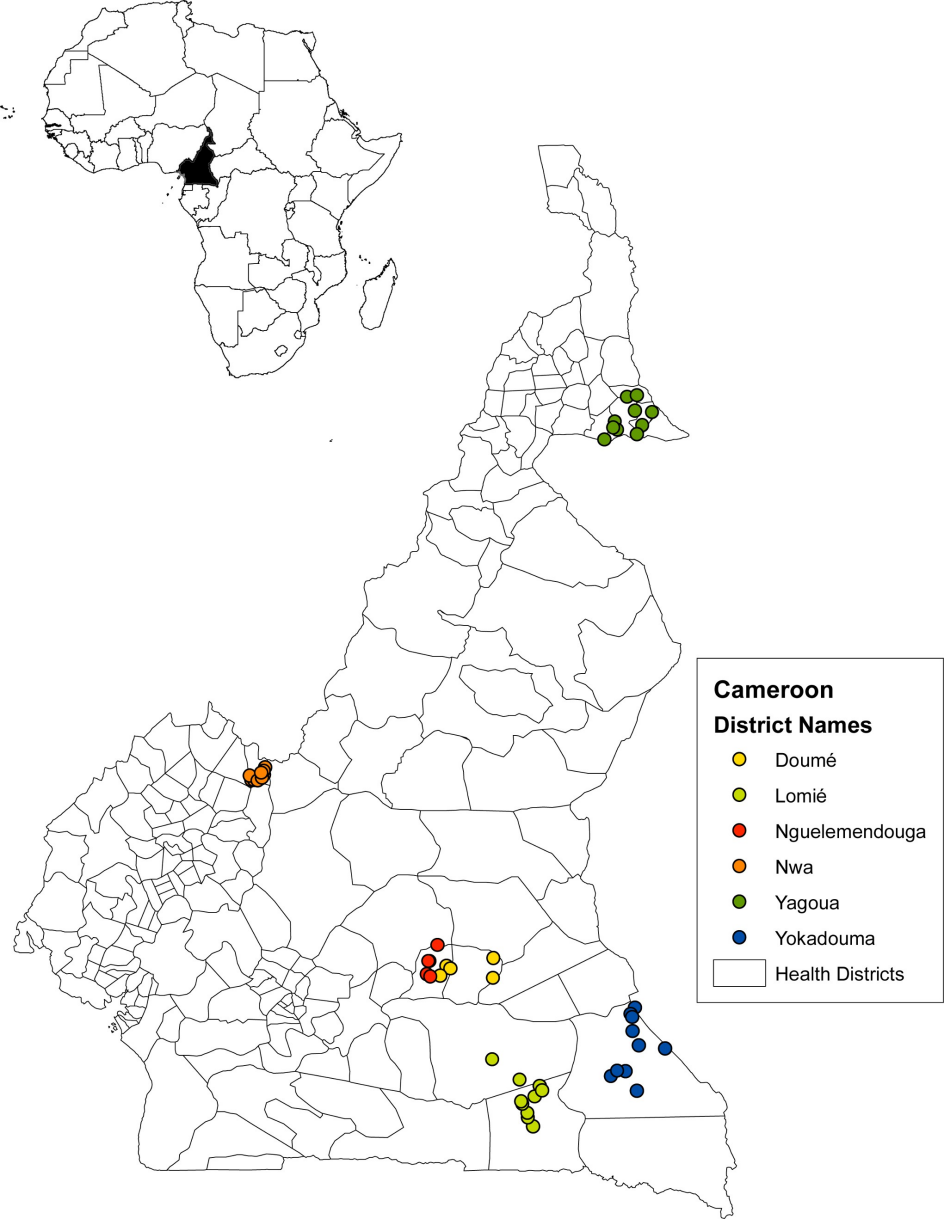
Map of Cameroon. This map shows each village location within the six managing health districts enrolled in the study. Lomie health district managed a village for the neighboring Abong Mbang health district. This map was created using ArcGIS (ArcMap v10.5.1) software by Esri.

In November and December of 2016 field data were collected from six health districts located in five distinct areas in four geographical regions/bioecological zones (Fig 1, Table 1). The map in Fig 1 was created using ArcGIS (ArcMap v10.5.1) software by Esri. ArcGIS and ArcMap are the intellectual property of Esri and are used herein under license. The shape files were generated in house. A total of 50 communities were selected: 10 communities per managing health district, with the exception of the Doume and Nguelemendouka health districts where 5 communities from each were enrolled. The different bioecological zones represented varying levels of endemicity of loaisis, circulating filarial antigen (ICT prevalence, potentially indicative of *W*. *bancrofti*) and other filarial parasites (*Onchocerca volvulus*, *Mansonella perstans*, *M*. *streptocerca*). The districts have varying histories of previous ivermectin treatment (Table 1). The Yagoua health district in the Far-North region is situated in the shrub steppes and was considered endemic for LF more than 30 years ago [6], but with no loiasis based on RAPLOA data [15]. The Nwa health district in the grassland savannah of the Northwest region showed low to moderate ICT rates with varying levels of *L*. *loa* prevalence [13]. In the eastern region, the Doume and Nguelemendouka health districts are part of the mosaic forest-savannah zone and the ICT prevalence rates were relatively high with varying prevalence of *L*. *loa* based on RAPLOA. In the east region, the Yokadouma and Lomie health districts were both located in the dense humid rainforest and had relatively high ICT and *L*. *loa* prevalence rates compared to the northern regions.

**Table 1:** Ecological properties and history of community-directed treatment with ivermectin (CDTi) of communities/villages screened for the presence of lymphatic filariasis in Cameroon. Health districts that were hyper- or mesoendemic for onchocerciasis received CDTi while districts hypoendemic for onchocerciasis received no CDTi.

Health District DiDistrict	Yagoua	Nwa	Nguelemendouga	Doumé	Yokadouma	Lomié
**Community number**	1	2	3	4	5	6
**Region**	Far Northern	Northwestern	Eastern (central)	Eastern (central)	Eastern	Eastern (south)
**Bioecological zone**	Shrub steppes	Forested savannah	Equatorial rainforest	Equatorial rainforest	Humid Equatorial rainforest	Humid Equatorial rainforest
**RAPLOA classification**	Non-endemic	High endemicity	High endemicity	High endemicity	High endemicity	High endemicity
**Ivermectin MDA status**	12 years annual CDTi	9 years annual CDTi	No CDTi	No CDTi	No CDTi	No CDTi

### Study design and population

A cross-sectional community-based study design was used and from each of the 50 villages 100 residents were selected according to the guidelines for rapid mapping of bancroftian filariasis in Africa [16]. Villages were randomly selected from a list of villages provided by the health center in the health district. If a village was inaccessible by car, a nearby village that was accessible was selected as replacement. Lomie health district managed some villages that were geographically located in the neighboring health district Abong Mbang but were difficult to access from the Abong Mbang health center. Therefore, one village located in Abong Mbang, but managed by Lomie was included (Fig 1).

**Fig 2:**
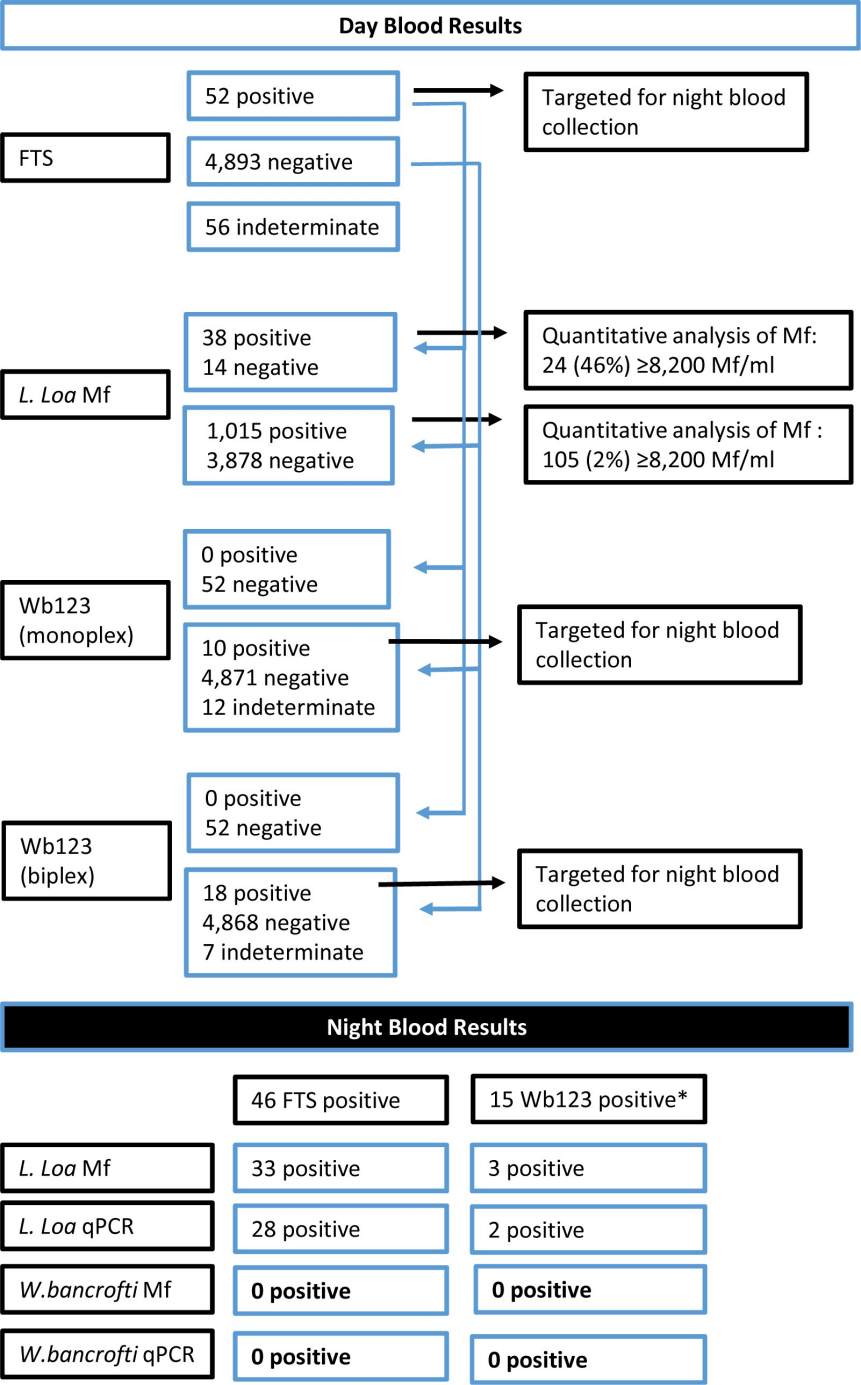
Study profile. Study profile showing the main study results. The rapid point of care tests FTS, Wb123 (monoplex) and Wb123 (biplex) sometimes produced indeterminate results. *positive by Wb123 monoplex or biplex test.

Capillary blood samples were collected from male and female study participants aged 10 years or older and tested for Mf in day blood, circulating filarial antigen and anti-filarial antibodies. Individuals that tested positive for circulating filarial antigen, or filarial antibodies were re-examined at night using thick blood films and qPCR to detect *W*. *bancrofti* Mf. The study profile is found in Fig 2

### Ethical considerations

This study received approval from the Cameroon National Ethics Committee (N^o^ 2016/12/835/L/CNERSH/SP) and administrative clearance from the Ministry of Public Health. Furthermore, the Institutional Review Board of Washington University School of Medicine in St. Louis approved the study. In the field, the objectives and procedures were explained to the community leaders and all study participants. Parent or guardian of any child participant provided informed consent on the child’s behalf. Participation was voluntary and any participant could withdraw at any moment during the study without obligations.

### Clinical examination

Trained medical personnel recruited for this study examined all the participants for lymphoedema. All individuals were examined for signs of the limb lymphedema and, if present, lymphedema severity was graded [17]. In addition, males were assessed for hydrocele.

### Detection of circulating filarial antigen and anti-filarial IgG4 antibodies

Circulating filarial antigen was detected using filariasis test strips (FTS, Alere Scarborough, Maine, USA). The FTS devices were stored at room temperature and carried to the field in cooled polystyrene foam boxes. In each community, blood from each eligible participant was tested directly in the field by FTS according to the manufacturer’s instructions. Briefly, 75μL of heparinized finger-prick blood collected between 10am and 3pm was applied to the sample application pad of the FTS. The reading of the results was performed strictly after 10 minutes. A single test was performed for each participant.

For the detection of anti-filarial IgG4 antibodies, two different rapid diagnostic tests, a monoplex and a biplex coupled with the Ov16 antigen reactive with *O*. *volvulus* antibodies were used according to the recommendation of the manufacturer (Standard Diagnostics, Yongin, South Korea) [18]. Both tests were performed in parallel with the FTS test on capillary day blood samples.

### Parasitological examination

Diurnal blood collections were performed between 10am-3pm while nocturnal collections were done between 10pm-12am. For diurnal collections, 50μL non-heparinized finger-prick blood was used to prepare thick blood films (TBF) for the detection of Mf. At night, two separate non-heparinized finger-prick blood samples were collected: one 50 μL sample was used to prepare a TBF and the other 60 μL sample was used to generate preserved dried blood spots on filter paper (TropBio, Townsville, Australia). The TBF was stained with 10% Giemsa using standard procedures [6] and examined using a light microscope at 100-fold magnification for any blood dwelling Mf. Mf were identified by species, quantified and recorded. To aid proper identification of Mf, slides were sometimes also examined at 400- or 1,000-fold magnification.

### Detection of filarial DNA in night blood by qPCR

Night blood was collected on filter paper from 61 subjects. DNA extractions were done from 60 μl of night blood, which was evenly distributed on filter paper disks, 10 μl on each “ear” (TropBio, Townsville, Australia). Briefly, all six disks were collected in a 1.5 ml tube and submerged in 200 μl of 1X PBS and 20 μl proteinase K. After incubation at 56°C for an hour the DNeasy blood and tissue kit (Qiagen, Hilden, Germany) was used to perform the remaining steps of the protocol. The final elution was performed in 100 μl DEPC water. Using TaqMan Master Mix (Applied Biosystems, Foster City, CA, USA) as reaction chemistry, samples were tested in two separate qPCRs for of *W*. *bancrofti* and *L*. *Loa*. For the detection of *W*. *bancrofti* the “long DNA repeat” (LDR) was used as a target with primers and probes as described previously [19]. The primers and probes used for the *L*. *loa* qPCR were described by Fink and co-workers [20]. All oligonucleotides were obtained by Integrated DNA Technologies (Skokie, IL, USA). The qPCR was performed at the University of Buea using the CFX Biorad thermocycler with a 10 μl/well set up. All reactions were carried out in duplicates and non-template control and a known positive sample were included in each run. Samples were considered to be positive if the cycle threshold value was < 40. For quality control, all samples were re-tested at Washington University School of Medicine in St. Louis using a Fast TaqMan Master Mix and a Quantstudio 6 thermocycler (Applied Biosystems).

### Data collection and statistical analysis

Demographic information was collected using the EpiInfo for Mobile Devices App (Centers for Disease Control and Prevention, Atlanta, GA) on a Samsung Galaxy tablet. This information was linked for each study participant to a unique barcode identifier. This barcode was also attached to the laboratory data collection forms and used to track study results. Data were transmitted via cloud to Washington University School of Medicine and imported to SPSS v20.0.0 (Armonk, NY: IBM Corp) for analysis. The geometric mean intensity (GMI) of Mf counts was calculated as antilog (∑log(x+1) /n), with "x" being the number of Mf per mL of blood in Mf positive individuals and "n" the number of Mf positive individuals examined. To estimate prevalence, we used a generalized linear mixed model (PROC GLIMMIX [SAS, 9.4]) with a logit linking function and a random effect to account for correlation among villages within a district. Additionally, we used a classification-tree analysis (PROC HPSPLIT [SAS, 9.4]) to determine the Mf density cut point that best splits the FTS outcome into groups, and estimated the prevalence for the groups below and above the cut point.

## Results

### Microfilariae in day blood

Only Mf of *L*.*loa* and *M*. *perstans* were detected in day blood (Table 2). Five of the six study health districts were found to be endemic for loiasis (Mf prevalence ≥1%) with the highest prevalence of *L*. *loa* Mf in the central-east region (Doume & Nguelemendouga) ranging from 19 to 87%, and the lowest in the north-west region (Nwa) ranging from 2 to 18% (Supplementary File 1). The savanna region in the far north (Yagoua) was not endemic for *L*. *loa* with only a single Mf positive subject. The highest geometric mean *L*. *loa* Mf density in infected individuals was observed in the south-east (Yokadouma) region with 957.4 Mf/ml and the maximum Mf count in a subject with 164,210 Mf/ml. In this area the geometric mean *L*. *loa* Mf density varied between the villages from 241 Mf/ml to 3,192 Mf/ml.

**Table 2:** Prevalence (with 95% confidence interval CI), geometric mean density (with standard deviation SD) of infected individuals, and maximum count of microfilaria (Mf) of *L*. *loa* and *M*. *perstans* in day blood in 6 health districts in Cameroon.

Health District	N	*L*. *loa*			*M*. *perstans*		
		Mf % (95% CI)	Mean Mf/ml (SD)	Max Mf/ml	Mf % (95% CI)	Mean Mf/ml (SD)	Max Mf/ml
Yagoua	1000	0.1 (0.0, 0.8)	30.00	30	0	0	0
Nwa	1000	7.0 (4.5, 10.8)	621.91 (7.3)	23320	0	0	0
Nguelemendouga	484	40.6 (22.8, 61.2	524.6 (8.5)	145060	1.4 (0.2, 8.6)	80.3 (6.8)	2760
Doumé	501	47.2 (22.7, 73.8)	572.3 (9.8)	101730	16.1 (6.1, 35.9)	168.4 (6.9)	24080
Yokadouma	1016	28.9 (24.4, 33.9)	957.4 (10.9)	164210	1.8 (1.0, 3.2)	47.4 (6.0)	3910
Lomié	1000	25.6 (18.1, 34.8)	146.3 (7.3)	28610	6.2 (3.1, 12.2)	47.5 (3.6)	2870
Total	5001	12.8 (8.1, 19.7)	462.1 (10.0)	164210	1.4 (0.8, 2.5)	87.5 (6.0)	24080

*M*. *perstans* was co-endemic in four of the six regions, but prevalence rates and density of infection was much lower (Table 2). The highest prevalence of *M*. *perstans* was found in the central-east region of Doumé with prevalence of 43.9% in one village. The highest geometric mean density in infected subjects was 164.6 Mf/ml also in Doume. In this health district, the person with the highest count of *M*. *perstans* Mf was observed with 24,080 Mf/ml. The small, non-sheathed Mf of *M*. *perstans* were easily differentiated from the larger, sheathed Mf of *L*. *loa*. No Mf of other filarial parasites (*W*. *bancrofti* or *O*. *volvulus*) that usually do not circulate in day blood were observed.

### Circulating filarial antigen and filarial-specific antibodies

Circulating filarial antigen measured by the FTS assay as an indicator for *W*. *bancrofti* infection was detected in all six study areas and the overall prevalence was 1.1% (Table 3). However, the FTS prevalence rates in three areas were below 1%, while in the Nguelemendouga, Doume, and Yokadouma health districts areas rates were between 1.6 and 2.2%. In these areas, 16 (80%) of the 20 villages had rates of 1% or above (Supplemental File 1). The highest rates of positive FTS test results were detected in 2 villages of the Yokadouma area with 5.5 and 4.2%. Overall 14% of the study villages accounted for 50% of the positive FTS results. These results demonstrate that subjects with positive FTS results were not evenly distributed in the 50 study villages, but concentrated in the southern rainforests, where high *L*. *loa* Mf rates were observed. Overall, of the 52 FTS positive subjects, 38 (73.1%) were also positive for *L*. *loa* Mf by thick day blood smear (Table 2). Among these 38 FTS and *L*. *loa* Mf positive individuals, 24 (63%) had *L*. *loa* Mf densities of 8,200 Mf/ml or above (Fig 2).

**Table 3:** Frequency of positive filarial antigen tests (FTS) by *L*. *loa* microfilaria (Mf) density in the 6 study health districts in Cameroon.

	Yagoua		Nwa		Nguelemendouga		Doumé		Yokadouma		Lomié		Total	
Mf/mL	N^a^	FTS+	N	FTS+	N	FTS+	N	FTS+	N	FTS+	N	FTS+	N	FTS+ (%)
**0**	999	5	916	5	283	1	262	0	718	1	714	2	3,892	14 (0.3)
**1–7,999**	1	0	66	0	176	1	194	4	227	2	251	0	915	7 (0.8)
**8,000–30,000**	0	0	10	2	14	6	25	5	48	10	11	1	108	24 (22.2)
**>30,000**	0	0	0	0	6	0	5	2	19	5	0	0	30	7 (23.3)
**Total**	1,000	5	992	7	479	8	486	11	1,012	18	976	3	4,945	52 (1.1)

^a^- Samples that did produce an indeterminate FTS result are not included in N

Specific antibodies reactive with the recombinant *W*. *bancrofti* antigen *Wb*123 were assessed by two different Point-of-Care (PoC) diagnostic tests: The *Wb*123 monoplex test detects antibodies reactive with the *Wb*123 antigen alone and the *Wb*123/*Ov*16 biplex test detects antibodies reactive with *Wb*123 and with the *O*.*volvulus* specific antigen *Ov*16. In total, 22 subjects were positive with any of these two Wb123 tests, but there was a considerable difference between both tests. The *Wb*123 monoplex detected antibodies reactive with Wb123 in 10 subjects while the Wb123 biplex detected antibodies in 18 subjects. Of the 18 positive biplex individuals, 8 of them were also positive by the monoplex test. The distribution of positive *Wb*123 results was relatively even among the study areas and villages. Apart from one village that had two positive individuals, all other 49 villages had 0 or 1 subject with a positive *Wb*123 result. None of the subjects with a positive FTS test result were positive with any of the Wb123 antibody tests.

Since the biplex test also measures antibodies reactive with *Ov*16, data on the distribution of onchocerciasis were collected. The rate of positive Ov16 tests was highly variable among the 6 study districts (Table 4). Yagoua, Nguelemendouga, Doume, and Yokadoume each had a positive rate of 1% or less. In contrast, Lomie had 7.6% positive tests and Nwa had 28.8% positive Ov16 tests. In the latter area, the rate of positive tests varied within the villages between 15.2 and 50%.

**Table 4:** Summary of the antigen (FTS) and antibody (Wb123 monoplex, Wb123/Ov16 biplex) serology results (95% confidence interval, CI) using in the 6 study areas in Cameroon.

	N^a^	FTS % (95% CI)^b^	Monoplex Wb123% (95% CI)	Biplex	
Health District				Wb123% (95% CI)	Ov16% (95% CI)
Yagoua	1000	0.3 (0.1, 1.4)	0	0.2 (0.0, 0.8)	0.1 (0.0, 0.8)
Nwa	1000	0.7 (0.3, 1.5)	0.1 (0.0, 0.8)	0.4 (0.0, 0.9)	28.2 (22.0, 35.4)
Nguelemendouga	484	1.7 (0.9, 3.2)	0.4 (0.1, 1.4)	0.4 (0.1, 1.4)	0.6 (0.2, 2.4)
Doumé	501	2.3 (1.2, 4.1)	0.4 (0.1, 1.6)	0.3 (0.0, 2.7)	0.8 (0.2, 3.6)
Yokadouma	1016	1.6 (0.8, 3.2)	0.2 (0.0, 0.8)	0.4 (0.2, 0.9)	0.1 (0.0, 0.7)
Lomié	1000	0.3 (0.1, 0.9)	0.3 (0.1, 0.9)	0.4 (0.2, 0.9)	7.4 (5.2, 10.3)
Total	5001	1.0 (0.7, 1.4)	0.2 (0.1, 0.4)	0.4 (0.2, 0.5)	2.0 (1.0, 3.7)

a Number of subjects that provided a specimen.

b No individual positive by FTS was also positive by Wb123

### Microfilariae and filarial DNA in night blood

Subjects with positive FTS or PoC Wb123 test (monoplex or biplex) results were followed up for night blood collections to screen for Mf and DNA of *W*. *bancrofti*. In total, 46 night blood samples of FTS positive subjects were screened and none of them were positive for *W*. *bancrofti* either by microscopy or by qPCR (Table 5). In contrast, 32 of these night blood samples were positive for Mf of *L*. *loa* by microscopy and 28 were positive for *L*. *loa* by qPCR. Similarly, among the 15 Wb123 positive subjects who provided a night blood sample, none were positive for *W*. *bancrofti* by either assay (Table 6).

**Table 5:** qPCR results of FTS positives who were followed up for night blood collection.

Health District	FTS+ tested^a^	*Loa* Mf + (%)	Max Mf/ml	*Wb* qPCR+	*L*. *loa* qPCR+ (%)^b^
**Yagoua**	5	0	0	0	0
**Nwa**	7	2 (28.6)	22,080	0	2 (100)
**Nguelemendouga**	6	6 (100)	23,100	0	5 (83.3)
**Doumé**	10	10 (100)	50,350	0	8 (80.0)
**Yokadouma**	15	14 (93.3)	164,210	0	12 (85.7)
**Lomié**	3	1 (33.3)	20,470	0	1 (100)
**Total**	46	33 (71.7)	164,210	0	28 (84.8)

^a^ Only FTS positive individuals that provided a night blood samples were subjected to qPCR

^b^ Percentage is derived from the number of Mf+ samples

**Table 6:** qPCR results of Wb123 positive subjects who were followed up for night blood collection.

Health District	Wb123+ tested^a^	*Loa* Mf + (%)^b^	*Wb* qPCR+	*L*. *loa* qPCR+ (%)^b^
**Yagoua**	0	0	0	0
**Nwa**	4	0	0	0
**Nguelemendouga**	2	2 (100)	0	0
**Doumé**	3	0 (0)	0	1 (33.3)
**Yokadouma**	2	0 (0)	0	0
**Lomié**	4	1 (25.0)	0	1 (25.0)
**Total**	15	3	0	2

^a^ Positive by either monoplex or biplex assays

^b^ Percentage is derived by number of Wb123+ samples

**Fig 3:**
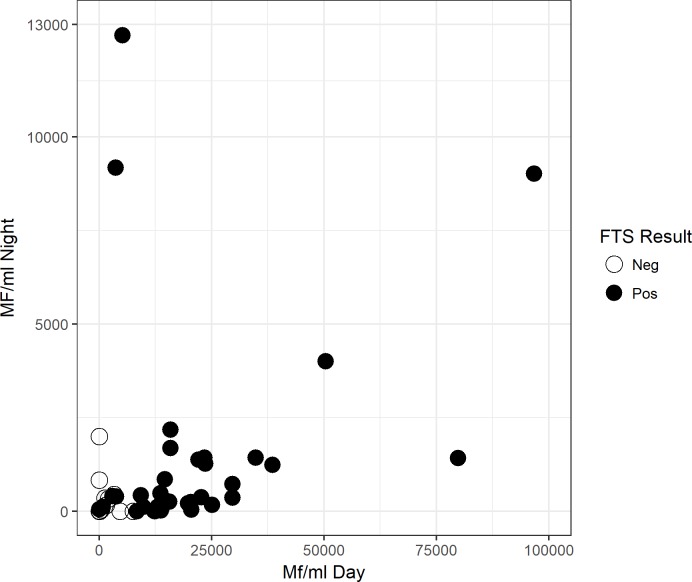
Comparison of microfilaria (Mf) densities in day and in night blood. Data were determined by thick blood smear of capillary blood. Samples were collected from subjects who were either circulating antigen (FTS) or antibody (Wb123) test positive during the initial screening.

The only Mf found in these 61 night blood samples where those of *L*. *loa*. In total 36 (60%) of these samples were positive for *L*.*loa* Mf, and the highest density of *L*.*loa* Mf in night blood was 9,180 Mf/ml. The densities of *L*. *loa* Mf in night blood were usually much lower compared to the densities of matching day blood samples (Fig 3).

### Strategy for LF mapping

Our data show that the FTS antigen test is highly specific in areas not endemic for loiasis, specifically Yagoua, where the *L*. *loa* Mf prevalence is less than 1%. In areas with higher loiasis prevalence, the PoC Wb123 tests can be used as confirmatory test, and thus far, it appears that biplex Wb123 test may have a higher sensitivity. If reliable rapid Wb123 diagnostic tests for confirmation are not available, examination of a thick blood smear of day blood collected from FTS positive individuals can be used as indicator for potential cross-reactivity with *L*.*loa* antigens. In order to determine the *L*. *loa* Mf density threshold at which positive FTS results should be excluded analysis and considered as indeterminate we performed a classification-tree analysis. An Mf density of 8,200 Mf/ml or less was determined as the cut point. At this density 0.4% (95% CI 0.3, 0.7) where FTS positive, while at higher densities (>8,200 Mf/ml) 22.5% (95% CI 16.8, 29.4) were FTS positive. Therefore, FTS results of subjects with more than 8,200 *L*. *loa* Mf per ml day blood during LF mapping should be considered as indeterminate. This adjustment would reduce in our case the sample size by only 0.6% and the overall rate of positive FTS results would drop by more than half from 1.0% to 0.4%. Furthermore, none of the individual health districts (implementation units) would have had an FTS rate of ≥1% and would require MDA to eliminate LF.

## Discussion

The present study showed that high densities of *L*. *loa* Mf confounds mapping of LF using FTS for the detection of circulating filarial antigen. This finding is in line with previous reports and indicates that tests for circulating filarial antigens alone cannot unambiguously detect LF endemicity is areas highly endemic for loiasis [11–13, 21]. These observations have profound implications for the classification of Cameroon as LF endemic country.

Our results of day blood examination showed that 5 of the 6 health district areas were endemic for *L*. *loa* and 4 of the 6 areas were endemic for *M*. *perstans* (Table 2). These results were expected and agree with previous reports [15, 22]. Although *L*. *loa* Mf densities were usually higher in day blood, some subjects had also relatively high Mf densities of >1,000 Mf/ml in night blood (Fig 2). This observation was reported previously but is still not widely recognized [11]. Furthermore, we found no evidence for the presence of *W*. *bancrofti* Mf by microscopy or qPCR in night blood (Tables 5 and 6). Therefore, it must be stressed that in loiasis endemic regions large, sheathed Mf in night blood may be due to *L*. *loa* and not due to *W*. *bancrofti* infection.

The rapid PoC Wb 123 monoplex and biplex tests are unlikely to cross-react with antibodies to *L*. *loa* or *M*. *perstans* and may be suitable as a confirmatory test for the FTS antigen detection. However, the detailed sensitivity and specificity of these tests in the field are not well established and the agreement was relatively poor. Therefore, we prefer to use *L*. *loa* Mf densities in day blood to validate FTS results. If FTS results of individuals with >8,200 *L*. *loa* Mf/ml are considered to be indeterminate and excluded from the analysis the problem of cross-reactivity is greatly reduced. Since usually only a small percentage of FTS tests are positive, determination of Mf density in day blood for these subjects should not be a logistical or technical problem. This can be easily done directly in the field by blood smear or if available by LoaScope technology [23].

The use of the Wb123/Ov16 biplex test also provided information about the distribution of onchocerciasis. A high rate of positive Ov16 results in the population does not necessarily mean that an area is still endemic for onchocerciasis and that transmission of *O*. *volvulus* is ongoing. However, a very low rate of positive Ov16 results (with the assumption of a decent sensitivity of the test device) indicates that the area is not highly endemic for onchocerciasis. Our results agree well with the known distribution of onchocerciasis in Cameroon with a major focus in the west and a smaller in the south [24].

Published information on the current or historical distribution of LF in Cameroon is very limited. The country has a population of about 23 million residents and few of them have been properly tested for LF. In a study performed from 2009 to 2010, 10,943 individuals from 8 regions were tested for circulating filarial antigen by ICT and positivity rates ranged from 1 to 8% [14]. In that study, positive tests were not confirmed by other tools to diagnose *W*. *bancrofti* infection and positive tests could be due solely to cross-reactivity with *L*. *loa* antigens. In a second survey, included in that study, Nana-Djeunga and co-workers tested more than 26,000 individuals from these regions for Mf in night blood and found zero Mf in 4 regions and less than 0.5% in the other regions [14]. Therefore, these mapping data do not support LF endemicity (infection prevalence ≥ 1%) in Cameroon.

A more recent study reported evidence of interruption of LF transmission in a few evaluation units in the North and Far North region [25]. Given the poor quality of mapping data, it is not clear whether these areas were endemic for LF before the start of MDA with ivermectin plus albendazole and whether MDA for LF elimination was justified. However, parts of these regions are endemic for onchocerciasis and received CDTi prior to MDA with ivermectin plus albendazole [26]. A recent literature review highlighted the very limited information available on LF prevalence in areas endemic for loiasis and found increasing evidence for low or no *W*. *bancrofti* prevalence in high risk *L*. *loa* areas [27]. Our study provides further evidence and proposes a solution for reliable verification mapping of LF in loiasis areas. Without any further LF specific intervention, it may be possible now to declare the entire country of Cameroon or at least large parts as non-endemic for LF.

In the present study we developed an algorithm to increase specificity of antigen testing using FTS for detection of *W*. *bancrofti* infection in areas endemic for *L*. *loa* by excluding FTS results from subjects with >8,200 *L*. *loa* Mf per ml. This threshold would also significantly reduce the number of FTS positive results because of cross-reactivity with *L*. *loa* from another study performed in Cameroon [12]. However, additional data would be helpful to determine the robustness of this threshold. Investing in proper mapping of LF in *L*. *loa* endemic areas may not only help to avoid unnecessary MDA for LF elimination, it may also help to shrink the map of LF distribution. It is possible that in Central Africa where loiasis is endemic, the GPELF is much closer to its goal than assumed previously.

## Conclusion

This study confirmed that high loads of *L*. *loa* Mf confounds LF mapping using circulating filarial antigen tests. Validation of FTS positive subjects using Wb123 tests or better tests for high *L*. *loa* Mf loads can help to circumvent the problem of cross-reactivity of antigen tests with *L*. *loa*. All FTS positive samples with high *L*. *loa* Mf loads (>8,200 Mf/ml) should be considered as indeterminate FTS results and should be excluded from the analysis.

## Supporting information

S1 File Village level test results. (XLSX)
